# Supplementary material for: Copy Number Alterations of Depressed Colorectal Neoplasm Predict the Survival and Response to Oxaliplatin in Proximal Colon Cancer
Source: Cancers (Basel). 2020 Jun 10;12(6):1527. doi: 10.3390/cancers12061527 (PMC7352996; doi:10.3390/cancers12061527)
Supplement: Supplementary file 1 [file cancers-12-01527-s001.pdf]

Supplementary Materials

Copy Number Alterations of Depressed Colorectal Neoplasm Predict the Survival and Response to Oxaliplatin in Proximal Colon Cancer

Li-Chun Chang, Han-Mo Chiu, Bing-Ching Ho, Min-Hsuan Chen, Yin-Chen Hsu, Wei-Tzu Chiu, Kang-Yi Su, Chia-Tung Shun, Jin-Tung Liang, Sung-Liang Yu and Ming-Shiang Wu

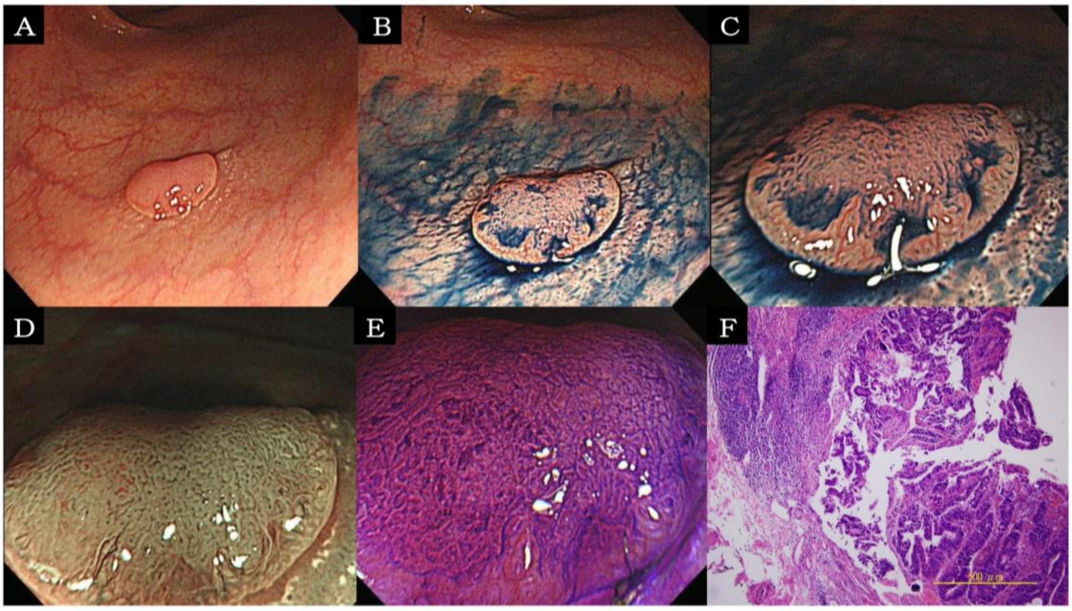

Figure S1. Depressed neoplasm has invasive cancer at small-size stage. (**A**) White light endoscopy: The 8-mm depressed morphology of the colorectal neoplasm located in the rectum. (**B**) Indigo carmine dye: The centrally depressed area contrasted out clearly after spraying it with the indigo carmine dye, and it corresponded with stage 0-IIa+IIc in morphology. (**C**) Indigo carmine dye: Closer examination showed that the region outside the demarcation line of the depressed area has a normal pit pattern. The sharp transition from normal mucosa to advanced histology directly was the distinct features in the carcinogenesis of the depressed colorectal cancer. (**D**) Narrow-band imaging: The narrow-band imaging disclosed that the vascularity of the depressed part was focally sparse. (**E**) Crystal violet stain: The pit pattern was irregular as observed after crystal violet staining (Vi pit pattern). (**F**) Histology: The histology revealed that the depressed region harbored invasive cancer with superficial submucosal involvement.

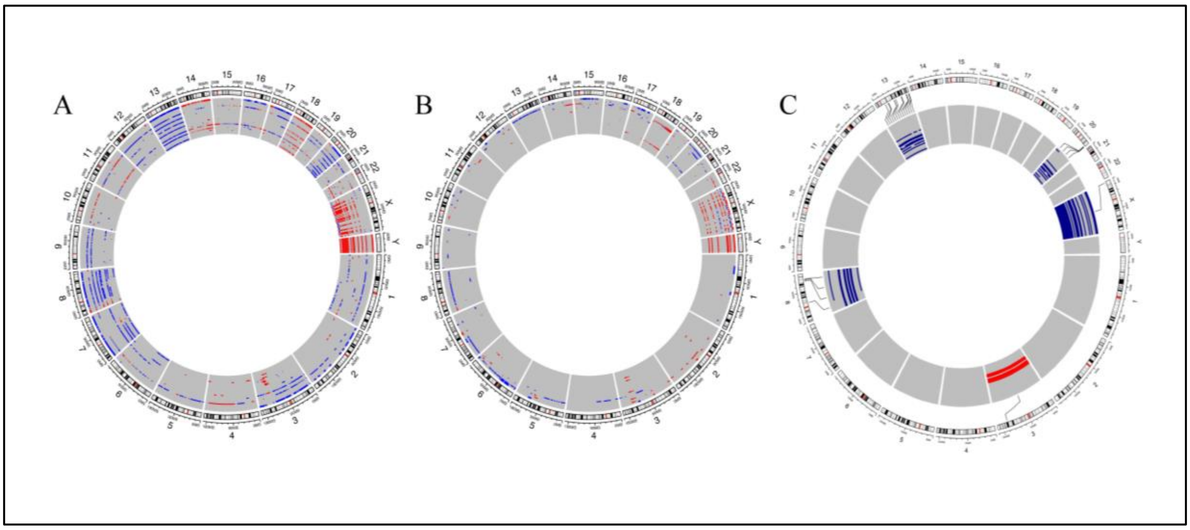

Figure S2. Copy number alterations in depressed and polypoid neoplasms. (**A**) Copy number alterations (CNAs) in depressed neoplasm. The blue color indicates CNA gain, and the red color indicates CNA loss. (**B**) CNAs in polypoid neoplasms. (**C**) Compared with polypoid neoplasms, depressed neoplasms have a CNA gain in chr8, chr13, chr20, and chrX and CNA loss in chr3.

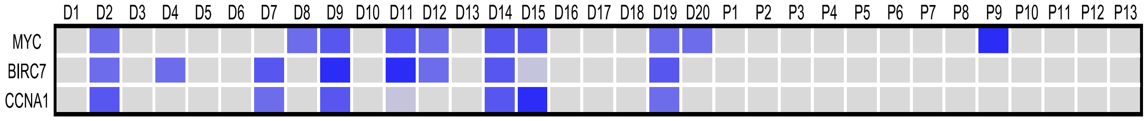

Figure S3. Arm-level changes in *MYC*, *CCNA1*, and *BIRC7* in each case in the discovery set.

Table S1. Matched protein coding genes with significant arm-level changes in chr8, chr13, and chr20.

Gene	Chr	Start	End
HAS2	chr8	122625270	122653630
ZHX2	chr8	123793900	123986755
DERL1	chr8	124025403	124054663
TBC1D31	chr8	124084858	124164392
FAM83A	chr8	124191286	124222319
C8orf76	chr8	124232195	124253638
ZHX1-C8orf76	chr8	124238428	124286727
ZHX1	chr8	124260689	124287781
ATAD2	chr8	124332090	124408705
WDYHV1	chr8	124428964	124460330
FBXO32	chr8	124510126	124553493
KLHL38	chr8	124657914	124665190
ANXA13	chr8	124693033	124749666
FAM91A1	chr8	124780678	124827690
FER1L6	chr8	124864226	125132302
TMEM65	chr8	125323158	125384940
TRMT12	chr8	125463047	125465266
RNF139	chr8	125487007	125500859
TATDN1	chr8	125500734	125551329
NDUFB9	chr8	125551342	125562227
MTSS1	chr8	125563010	125740748
ZNF572	chr8	125985538	125991630
SQLE	chr8	126010719	126034525
NSMCE2	chr8	126104082	126379367
TRIB1	chr8	126442562	126450647
FAM84B	chr8	127564682	127570711
PCAT1	chr8	128025398	128033259
PCAT2	chr8	128084938	128094466
PRNCR1	chr8	128092118	128104840
CASC19	chr8	128200030	128209872
CCAT1	chr8	128219626	128231513
CASC21	chr8	128256881	128404876
CASC8	chr8	128301920	128494384
CCAT2	chr8	128412643	128414395
POU5F1B	chr8	128427856	128429441
CASC11	chr8	128712852	128746213
MYC	chr8	128748314	128753680
PVT1	chr8	128806778	129113499
TMEM75	chr8	128958804	128960969
USP12	chr13	27640286	27746033
RPL21	chr13	27825691	27830702
RPL21P28	chr13	27825692	27830699
RASL11A	chr13	27844463	27847827
GTF3A	chr13	27998680	28009846
MTIF3	chr13	28009775	28024739
LNX2	chr13	28120049	28194720

POLR1D	chr13	28194879	28241559
GSX1	chr13	28366779	28368089
PDX1	chr13	28494167	28500451
ATP5EP2	chr13	28519342	28519710
CDX2	chr13	28536204	28543505
URAD	chr13	28552242	28562774
FLT3	chr13	28577410	28674729
PAN3	chr13	28712642	28869475
CCDC169	chr13	36801178	36871992
SPG20	chr13	36875774	36944317
CCNA1	chr13	37005966	37017019
SERTM1	chr13	37248048	37271975
RFXAP	chr13	37393338	37403740
SMAD9	chr13	37418967	37494409
ALG5	chr13	37523907	37573504
EXOSC8	chr13	37574677	37583751
SUPT20H	chr13	37583450	37633850
CSNK1A1L	chr13	37677396	37679801
POSTN	chr13	38136718	38172981
TRPC4	chr13	38210772	38443939
UFM1	chr13	38923907	38937144
FREM2	chr13	39261172	39461267
STOML3	chr13	39540061	39564996
PROSER1	chr13	39584001	39612213
NHLRC3	chr13	39612447	39624244
LHFP	chr13	39917028	40177356
COG6	chr13	40229763	40365802
SLITRK5	chr13	88324869	88331870
DAOA	chr13	106118215	106143383
SPACA7	chr13	113030650	113089009
TUBGCP3	chr13	113139318	113242499
ATP11AUN	chr13	113301357	113338811
ATP11A	chr13	113344642	113541482
MCF2L	chr13	113623534	113754053
F7	chr13	113760101	113774995
F10	chr13	113777112	113803843
PROZ	chr13	113812967	113826698
PCID2	chr13	113831852	113863234
CUL4A	chr13	113862506	113919392
LAMP1	chr13	113951468	113977741
GRTP1	chr13	113978478	114018463
ADPRHL1	chr13	114076254	114107839
DCUN1D2	chr13	114110133	114145023
TMCO3	chr13	114145307	114204544
TFDP1	chr13	114239002	114295788
ATP4B	chr13	114303121	114312513
GRK1	chr13	114321596	114438637
TMEM255B	chr13	114462215	114514899
GAS6	chr13	114523521	114567076

RASA3	chr13	114747193	114898098
CDC16	chr13	115000319	115038201
UPF3A	chr13	115047058	115071291
CHAMP1	chr13	115079809	115092803
TAF4	chr20	60549853	60640866
LSM14B	chr20	60697516	60710434
PSMA7	chr20	60711782	60718514
SS18L1	chr20	60718775	60757566
MTG2	chr20	60758080	60777810
HRH3	chr20	60790016	60795323
OSBPL2	chr20	60813540	60871269
ADRM1	chr20	60877951	60883918
LAMA5	chr20	60884115	60942368
RPS21	chr20	60962120	60963576
CABLES2	chr20	60963685	60982339
RBBP8NL	chr20	60985292	61002629
GATA5	chr20	61038552	61051026
SLCO4A1	chr20	61273796	61303647
NTSR1	chr20	61340188	61394123
MRGBP	chr20	61427756	61433090
OGFR	chr20	61436176	61445352
COL9A3	chr20	61448413	61472511
TCFL5	chr20	61472365	61493115
DPH3P1	chr20	61475917	61477543
DIDO1	chr20	61509089	61569304
GID8	chr20	61569440	61579827
SLC17A9	chr20	61583998	61599949
BHLHE23	chr20	61637330	61638387
HAR1B	chr20	61726844	61733671
HAR1A	chr20	61732643	61735737
YTHDF1	chr20	61826781	61847538
BIRC7	chr20	61867234	61871859
NKAIN4	chr20	61872135	61885892
FLJ16779	chr20	61885329	61892967
ARFGAP1	chr20	61904136	61921142
COL20A1	chr20	61924537	61962285
CHRNA4	chr20	61974661	61992748
KCNQ2	chr20	62031566	62104030
EEF1A2	chr20	62119364	62130668
PPDPF	chr20	62152132	62153524
PTK6	chr20	62159775	62168723
SRMS	chr20	62171276	62178857
HELZ2	chr20	62189438	62205592
GMEB2	chr20	62218946	62258454
STMN3	chr20	62271057	62284963
RTEL1	chr20	62289162	62327606
RTEL1-TNFRSF6B	chr20	62289162	62330051
TNFRSF6B	chr20	62328003	62330051
ARFRP1	chr20	62329994	62339365

ZGPAT	chr20	62338793	62367494
LIME1	chr20	62367052	62370460
SLC2A4RG	chr20	62371210	62375403
ZBTB46	chr20	62375020	62436856
ABHD16B	chr20	62492565	62494341
TPD52L2	chr20	62496580	62522898
DNAJC5	chr20	62526454	62567384
UCKL1	chr20	62571181	62587800
ZNF512B	chr20	62588056	62601223
SAMD10	chr20	62605465	62610995

Table S2. Diagnostic performance of the D-mark panel for detecting depressed neoplasms.

	**Discovery Set**		
	**Depressed neoplasm**	**Polypoid neoplasm**	
D-marker panel (+)	11	1	PPV (95%CI):<br>91.7% (65.8-99.6%)
D-marker panel (-)	9	12	NPV (95%CI):<br>57.1% (42.3-61.9%)
	Sensitivity (95%CI):<br>55.0% (39.5-59.7%)	Specificity (95%CI):<br>92.3% (68.4-99.6%)	Accuracy (95% CI):<br>69.7% (50.9-75.4%)
	**Validation set**		
	Depressed neoplasm	Polypoid neoplasm	
D-marker panel (+)	28	7	PPV (95%CI):<br>80.0% (62.5-90.9%)
D-marker panel (-)	9	35	NPV (95%CI):<br>79.5% (64.2-89.7%)
	Sensitivity (95%CI):<br>75.7% (58.4-87.6%)	Specificity (95%CI):<br>83.3% (68.0-92.5%)	Accuracy (95% CI):<br>79.7% (69.2-88.0%)

© 2020 by the authors. Licensee MDPI, Basel, Switzerland. This article is an open access article distributed under the terms and conditions of the Creative Commons Attribution (CC BY) license (http://creativecommons.org/licenses/by/4.0/).
